# Research, Innovation, and National Production of Strategic Inputs for Tuberculosis Elimination in Brazil: Contributions from the REDE-TB

**DOI:** 10.1590/0037-8682-0518-2023

**Published:** 2023-12-01

**Authors:** Ricardo Alexandre Arcêncio, Erica Chimara, José Roberto Lapa e Silva, Julio Croda, Anna Cristina Calçada Carvalho

**Affiliations:** 1 Escola de Enfermagem de Ribeirão Preto da Universidade de São Paulo, Ribeirão Preto, SP, Brasil.; 2 Instituto Adolfo Lutz, São Paulo, SP, Brasil.; 3 Universidade Federal do Rio de Janeiro, Rio de Janeiro, RJ, Brasil.; 4 Universidade Federal de Mato Grosso do Sul, Faculdade de Medicina, Campo Grande, MS, Brasil.; 5 Instituto Oswaldo Cruz, Rio de Janeiro, RJ, Brasil.

The Member States of the United Nations formally adopted the Political Declaration of the High-Level Meeting on Tuberculosis (TB) Elimination at the 78^th^ United Nations General Assembly held in New York on September 22, 2023^1^. Among the various points highlighted in the Declaration, we emphasize the reaffirmation of the collective commitment to the 2030 Agenda and the Sustainable Development Goals (SDGs), SDG 3.3 in particular, which emphasizes the need to end TB as an epidemic and the importance of interacting with other SDGs for poverty eradication (SDG 1) and the reduction of social inequalities (SDG 10)^1^.

Moreover, the Declaration underscores the importance of advancing TB prevention through screening and preventive treatment, preventing infection in at-risk populations, and highlighting the need for an effective and long-lasting TB vaccine. Accelerated investment in vaccine development and TB research funding are included and extensively highlighted throughout the document. With regard to financing in particular, the Member States are expected to commit to the costs of developing inputs and technologies that ensure equal access to health, dignified and respectful treatment, and robust innovative technologies for accurate diagnosis, including the detection of *Mycobacterium tuberculosis* drug resistance, drug development, vaccines, and outcome assessment technologies^2^.

Although the Declaration did not explicitly address the issue of sovereignty and autonomy of the Member States in the development of their diagnostic technologies, drug production, and vaccines, countries such as Brazil have accumulated considerable expertise in vaccine development, production of blood derivatives, and strategic health inputs throughout their history in public health. Substantial milestones include the production of generic drugs, patent breaking, and antiviral production for HIV/AIDS treatment, which have reduced dependence on imports and allowed for more affordable prices. However, owing to the lack of a state policy in recent years that generates competitiveness for national products to be incorporated into the Unified Health System, a more assertive and strategic position by governments and authorities is needed.

It becomes urgent to Adopting more robust and lasting financing mechanisms for projects is the immediate need to ensure the independence of national industries in the production of strategic inputs. The recent experience with COVID-19, wherein industrialized countries adopted restrictive measures on the export of inputs related to vaccines, medicines, and medical equipment as a pandemic response, revealed our continued vulnerability^3^. Because we are lacking in technology for vaccine production, approximately 50% of the national immunization program's budget is dedicated to acquiring mRNA platform vaccines for COVID-19, which is yet another clear example of our limited technological autonomy in healthcare.

**FIGURE 1:**
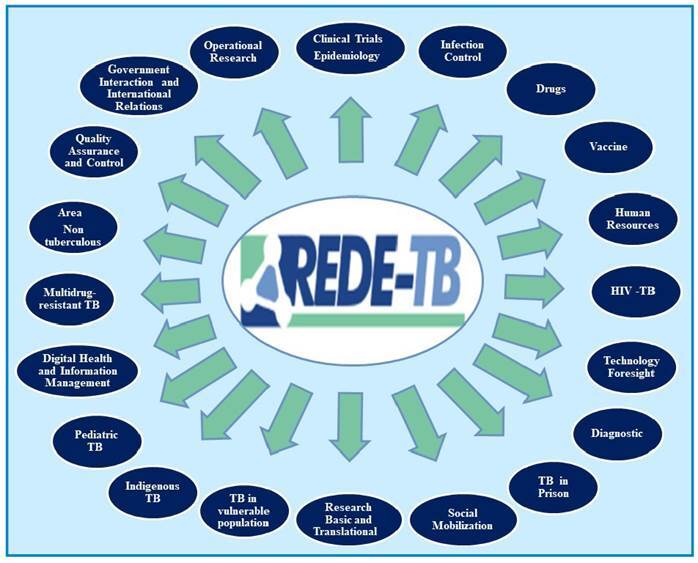
Research/Operational Areas of REDE-TB.

Currently, Brazil has a legal framework that allows the adoption of innovations^4^. Despite the existing laws and decrees that allow for the incorporation of these innovations, most managers are still unaware of these laws, leading to legal uncertainty that can result in an innovation not being properly incorporated^5^. This legal uncertainty hampers research, development, and state autonomy. However, public policies encouraging the application of these laws are also needed^6^. Therefore, the Brazilian Tuberculosis Research Network (REDE-TB, Figure 1), which is responsible for 80% of the scientific and technological production in the field of TB in Latin America, comprehends the need for more directive policies to ensure autonomy and national self-sufficiency in the production of strategic inputs to achieve TB elimination.

Despite the country's advanced capacity to produce a purified protein derivative (PPD) for the tuberculin test (which was lacking in the Unified Health System), the BCG vaccine (which also faced stockouts), medicines, and diagnostic technologies, inductive measures are still required to motivate researchers and valorize their products, allowing for the highest level of innovation capacity in health and strengthening sovereignty in the production and purchase of inputs, thereby ensuring the independence of the Unified Health System in the face of international market fluctuations and external pressures.

Governments that have increased their investments in research and development (R&D), technology, education, and infrastructure with improvements in regulatory environments and tax incentives have been able to quickly overcome periods of economic crisis, confirming the role of innovation as a key element of global competitiveness^7^.

In April 2023, the recent establishment of the Interministerial Committee for the Elimination of Tuberculosis and Other Socially Determined Diseases (CIEDDS)^8^ and a new strategy for the country's reindustrialization, with the launch of the National Strategy for the Development of the Health Economic-Industrial Complex in September 2023, with an expected investment of R$ 42 billion by 2026^9^, ensured that the commitments made by Brazil in the Political Declaration of the High-Level Meeting on TB Elimination will not remain on paper.
